# Uretero-Internal Pudendal Artery Fistula with Longterm Indwelling of Ureteral Stent: A Case Report

**DOI:** 10.1155/2012/817942

**Published:** 2012-06-03

**Authors:** Hideo Yuki, Yasuhiro Takayama, Masahisa Takuma, Mika Takahashi, Tadasuke Ando, Yasuhiro Sumino, Takeo Nomura, Fuminori Sato, Hiromitsu Mimata

**Affiliations:** ^1^Department of Urology, Nankai Hospital, 11-20 Tokiwanishi-machi, Saiki 876-0857, Japan; ^2^Department of Radiology, Nankai Hospital, Saiki 876-0857, Japan; ^3^Department of Urology, Faculty of Medicine, Oita University, 1-1 Idaigaoka, Hasama-machi, Yufu 879-5593, Japan

## Abstract

A 74-year-old woman presenting with bilateral ureteral stricture was referred to our hospital. She had undergone radical hysterectomy and adjuvant irradiation therapy for cervical cancer in 2000. Double-J stents were inserted in both the ureters and replaced at regular intervals. Eighteen months after ureteral stenting, she complained of gross hematuria and was managed with hemostatic agents. During a routine replacement of the right double-J stent, massive bleeding was observed from the urethra which continued intermittently. The source of bleeding was not identified on computed tomography and angiography. We kept her at rest, which reduced the bleeding. However, she required intermittent transfusions. Angiography was performed at the time of bleeding on March 5, 2011. A uretero-internal pudendal artery fistula was found, and coil embolization was performed. Thereafter, hematuria did not recur up to the last followup in July 2011.

## 1. Introduction

Ureteroarterial fistula is a rare condition that often causes life-threatening bleeding [1–9]. Previous pelvic surgery, radiation therapy, and indwelling of ureteral stents are closely associated with the development of this fistula [1–4]. Ureteroarterial fistula has been mainly treated by either surgery or a combination of surgery and arterial embolization [1, 5]. In this report, we present a patient with ureterointernal pudendal artery fistula who was successfully treated with coil embolization.

## 2. Case Report

The patient was a 74-year-old woman who had undergone radical hysterectomy and radiation therapy for cervical cancer in 2000. She was never followed up by urologists for postoperative neurogenic bladder. In April 2009, she was referred to our hospital because of acute renal failure with bilateral hydronephrosis. Her general status improved after indwelling of stents in both ureters, which were replaced every 2-3 months. Retrograde pyelography (RP) was performed at every instance of stent replacement. RP showed atrophic bladder and stenosis of the lower part of the ureters; however, there were no signs of a tumor. She complained of gross hematuria in October 2010, and cystoscopy showed bleeding from the right ureteral orifice. Computed tomography (CT) did not reveal the source of bleeding, such as, ureteral or pelvic cancer. The bleeding resolved spontaneously after 1 month of conservative therapy. Massive bleeding suddenly recurred during a routine replacement of the stents in December 2010, and blood transfusion became necessary.

**Figure 1:**
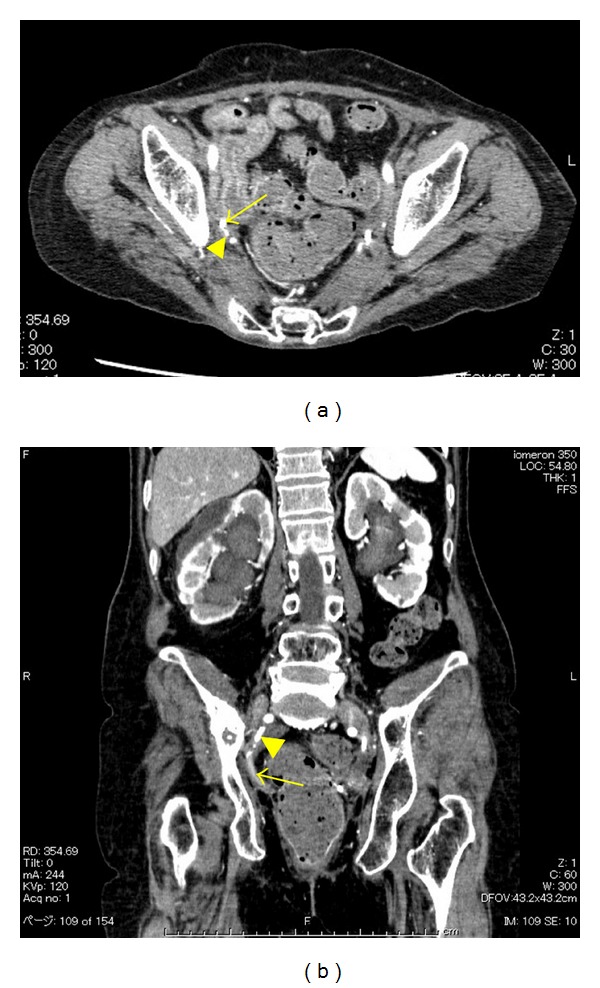
(a) Enhanced computed tomography scan showing the right ureter (arrow) and the right internal pudendal artery (arrowhead). (b) Three-dimensional computed tomography showing the right ureter (arrow) and the right internal pudendal artery (arrowhead).

**Figure 2:**
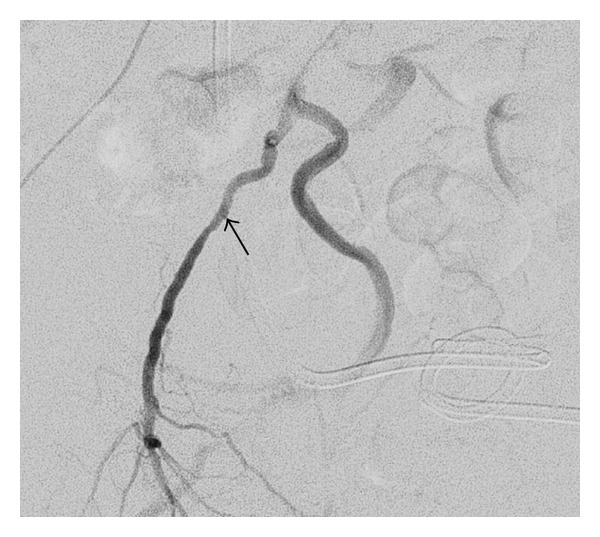
An angiogram showing no extravasation or fistula. However, a very small pseudoaneurysm (arrow) was seen retrospectively.

**Figure 3:**
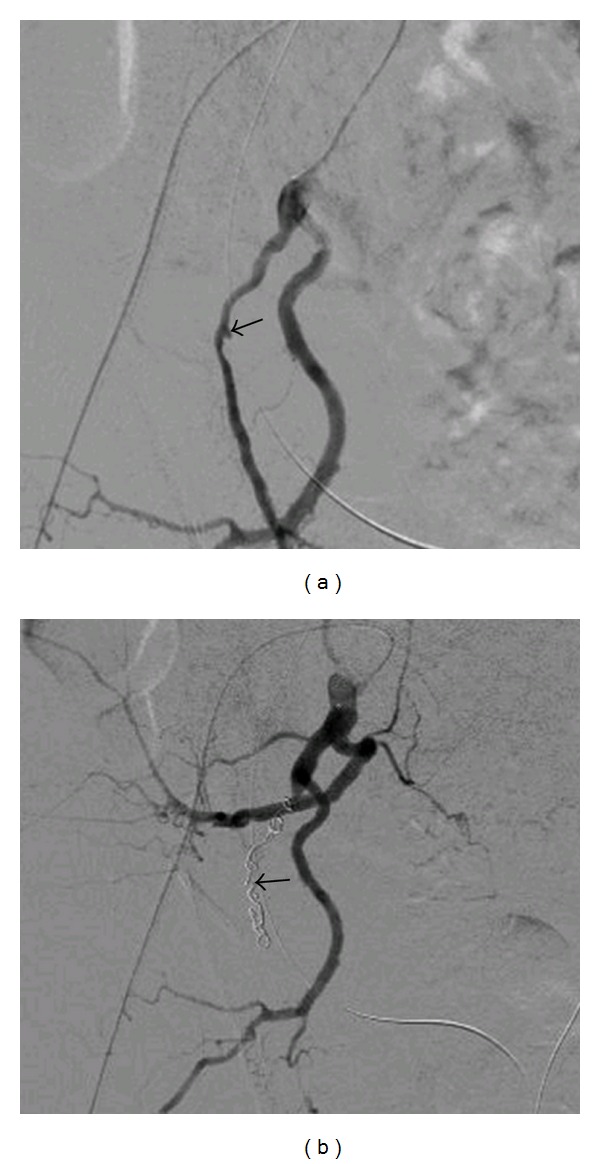
(a) A pseudoaneurysm (arrow) detected at the time of the second angiography. (b) Endovascular coils (arrow) inserted into the right internal pudendal artery.

Contrast-enhanced CT showed hematomas in the right renal pelvis and the ureter; however, there were no aneurysms or extravasations (Figures 1(a) and 1(b)). Angiography was performed, but no pseudoaneurysms were found, and the source of bleeding was not identified (Figure 2). Acute pyelonephritis was also observed. However, the infection and the hematuria were again resolved by conservative therapy. Massive bleeding recurred in March 2011. An immediate angiography was performed, which showed a fistula between the ureter and a pseudoaneurysm of the internal pudendal artery (Figure 3(a)). Coil embolization was performed (Figure 3(b)), which successfully controlled the bleeding. To avoid further indwelling of the ureteral stents, we recommended the creation of a urinary diversion; however, the patient refused to undergo any more open abdominal surgeries. After 3 weeks, nephrostomies were performed in both kidneys, and the ureteral stents were removed. Thereafter, hematuria did not recur up to the last followup in July 2011.

## 3. Discussion

Ureteroarterial fistulas mostly occur between the ureter and the ipsilateral iliac artery [1–9]. They have been reported to be associated with several conditions, including previous pelvic surgery, radiation therapy, indwelling of ureteral stents, infection, primary vascular disease, and pregnancy, with the first 3 being the most important [5, 6]. These conditions induce tissue fibrosis. The physical stimuli from ureteral stents with arterial pulsation may weaken the walls of both the artery and the ureter [3], and the fistula is formed consequently.

Ureteroarterial fistula can potentially result in life-threatening hemorrhage. Therefore, the presence of the fistula should be detected as soon as possible, particularly for patients who have undergone pelvic surgery, radiation therapy, and the ureteral stent insertion. To stop the hemorrhage, the source should be accurately identified, and three-dimensional CT or multiplanar reconstruction CT should be conducted [3]. However, finding the definite source of bleeding can be difficult because hemorrhage from fistula often occurs intermittently. When extravasation of vascular contrast medium is not detected by any imaging procedure, it is important to carefully look for pseudoaneurysms at the crossing site of the ureter on the radiographic images. At the same time, the presence of other diseases that may result in massive hemorrhage should also be considered. In our case, no pseudoaneurysms were detected at the time of the first angiography when the massive hemorrhage was resolved. However, angiography during the second massive hemorrhage showed a pseudoaneurysm. In addition, the identification of a fistula may be difficult when the bleeding point is unusual, as in our case. In most cases, fistulas form between the ureter and the common iliac artery at the crossing site [1, 2]. To the best of our knowledge, this is the first reported case in the literature of a fistula that formed between the ureter and the internal pudendal artery. The internal pudendal artery originates from the internal iliac artery and goes behind the ureter to the area of the rectum and hip. This artery is near the ureter but usually does not form a crossing site with it. Pelvic surgery causes deviation of both the ureter and the pudendal artery, and postoperative irradiation induces inflammation and fibrosis, which might firmly fix the ureter to the vessel, resulting in fistula formation. It is important to carefully observe the common artery as well as the peripheral artery, particularly in patients who have undergone pelvic surgery and radiation therapy.

If the fistula is not identified in the angiogram, removal of the stent to induce bleeding and finding the source of bleeding may be necessary [5].

Because ureteroarterial fistula is a life-threatening disease, identifying the point of fistula formation is of utmost importance. To stop the bleeding, a combination therapy involving intervention plus urinary diversion or intervention plus vascular graft implantation may be important [10].
